# Association between Syncope and the 6-Month Incidence of Ischemic Stroke, Arrhythmia, Brain Tumor, Epilepsy, and Anxiety Disorder

**DOI:** 10.3390/healthcare11131913

**Published:** 2023-07-02

**Authors:** Danilo Christian Gümbel, Christian Tanislav, Marcel Konrad, Louis Jacob, Ai Koyanagi, Lee Smith, Karel Kostev

**Affiliations:** 1 University Clinic, Philipps-University, 35043 Marburg, Germany; 2 Department of Geriatrics and Neurology, Diakonie Hospital Jung Stilling, 57074 Siegen, Germany; 3 Department of Health and Social, FOM University of Applied Sciences for Economics and Management, 60549 Frankfurt am Main, Germany; 4 Department of Physical Medicine and Rehabilitation, Lariboisière-Fernand Widal Hospital, AP-HP, University Paris Cité, 75010 Paris, France; louis.jacob.contacts@gmail.com; 5 Epidemiology of Ageing and Neurodegenerative Diseases (EpiAgeing), Inserm U1153, Université Paris Cité, 10 Avenue de Verdun, 75010 Paris, France; 6 Research and Development Unit, Parc Sanitari Sant Joan de Déu, CIBERSAM, ISCIII, Dr. Antoni Pujadas, 42, Sant Boi de Llobregat, 08830 Barcelona, Spain; 7 Institució Catalana de Recerca i Estudis Avançats (ICREA), Pg. Lluis Companys 23, 08010 Barcelona, Spain; 8 Centre for Health, Performance and Wellbeing, Anglia Ruskin University, Cambridge CB1 1PT, UK; 9 Epidemiology, IQVIA, 60549 Frankfurt, Germany

**Keywords:** syncope, stroke, cardiac arrhythmia, brain tumors, epilepsy, anxiety disorder, cohort study, Germany

## Abstract

Objectives: the aim of the present study is to investigate the associations between syncope and subsequent diagnoses of brain tumor, cardiac arrhythmia, stroke/transient ischemic attack (TIA), epilepsy, and anxiety disorder in a large outpatient population in Germany. Methods: This retrospective cohort study uses data from the Disease Analyzer database (IQVIA). Adults who received syncope diagnosis from one of 1284 general practices between January 2005 and December 2021 (index date) were included and matched (1:1) to individuals without syncope diagnosis using a propensity score based on age, sex, the number of consultations during the follow-up period (up to 6 months), and defined co-diagnoses documented within 12 months prior to and on the index date. Finally, associations between syncope and subsequent outcome diagnoses were investigated using multivariable logistic regression models. Results: Data related to 64,016 patients with and 64,016 patients without syncope (mean age 54.5 years, 56.5% female) were available. In total, 6.43% of syncope patients and 2.14% of non-syncope patients were diagnosed with one of the five outcome diagnoses within 6 months of the index date. There was a positive and significant association between syncope and incidences of ischemic stroke/TIA (OR = 2.83, 95% CI = 2.41–3.32), arrhythmia (OR = 3.81, 95% CI = 3.44–4.18), brain tumor (OR = 4.24, 95% CI = 2.50–7.19), epilepsy (OR = 5.52, 95% CI = 4.27–7.14), and anxiety disorder (OR = 1.99, 95% CI = 1.79–2.21). Conclusions: Syncope is significantly associated with an increased risk of subsequent ischemic stroke/TIA, cardiac arrhythmia, brain tumor, epilepsy, and anxiety disorder. Nevertheless, the cumulative incidences for all five diagnoses are very low.

## 1. Introduction 

Syncope is a sudden and temporary loss of consciousness (TLOC) due to a transient global cerebral hypoperfusion that is characterized by an immediate and complete recovery, usually within 10–30 s [1,2].

The causes of syncope are varied, ranging from often benign causes to severe disease patterns [1,3,4,5,6]; the most common groups of causes are as follows: neurally mediated reflex syncope, syncope due to orthostatic hypotension, and syncope due to cardiac arrhythmia [1].

With a lifetime cumulative incidence of up to 35% [7], hospitalization rates for those with the condition are reported to be vary, with rates of 38% [8], 46% [9] and 57.6% [10] recorded in past studies, and estimated annual costs for syncope-related admissions total 2.4 billion USD in the USA alone [11]. Clearly, therefore, syncope is of enormous medical and socioeconomic relevance. 

However, the cause of syncope remains unexplained in a significant number of cases, ranging from 16% to 36.6% [12,13,14,15] of cases, with diagnostic success depending to a significant extent on the thorough implementation of current diagnostic guidelines [13,16].

However, it is known that syncope can occur as a symptom or early sign of a variety of medical conditions, such as brain tumors, cardiac arrhythmia, transient ischemic attack (TIA), and epilepsy [1,17,18,19,20,21,22,23,24,25], and can also be associated with a variety of psychiatric disorders, most notably depression and anxiety disorders [26,27,28,29,30]. 

For example, 4.9% of 772 acute stroke/TIA patients included in a three-year study conducted by Ryan et al. reported syncopal or pre-syncopal symptoms at stroke onset. Of these patients, over 90% had a history of pre-syncopal symptoms and over 60% had a history of syncope [18]. 

Furthermore, Ungar et al. reported that syncope and epilepsy coexisted in approximately 60% of all patients with drug-resistant epilepsy, as well as approximately 20% of all patients with possible epilepsy, in their study [23].

In a meta-analysis performed by D’Ascenzo et al., which included 11 studies, 11% of patients who presented to the emergency department with syncope had a history of arrhythmic heart disease [14], while a multicenter prospective cohort study showed that of all patients who presented to emergency departments with syncope within 24 h of onset, nearly 2% returned and presented with cardiac arrhythmia within a 30-day period [29].

In a study conducted in 2022 that investigated the relationship between the number of total syncope episodes (TSE) and psychological status, Atici et al. showed a significant positive correlation between TSE and the Beck anxiety scale [28].

Although there have been many studies over time on the prognostics and prognostic stratification of syncope in the general population [31,32,33,34,35], there is a lack of studies that have examined the impact of syncope on the risk of developing the aforementioned diseases. 

Therefore, the aim of the present study is to investigate the associations between syncope and subsequent diagnoses of brain tumor, cardiac arrhythmia, stroke/TIA, epilepsy, and anxiety disorder in a large outpatient population in Germany.

## 2. Materials and Methods 

### 2.1. Database

Data from the Disease Analyzer database (IQVIA) was used to conduct the present retrospective study. The methodology of this database was previously detailed in the literature [36]. In brief, this database contains diagnoses, prescriptions, and demographic data collected in anonymized format from approximately 3% of general and 6% of specialized private practices in Germany. The database was considered representative of office-based practices operating in this country [36].

### 2.2. Study Population 

**Figure 1:**
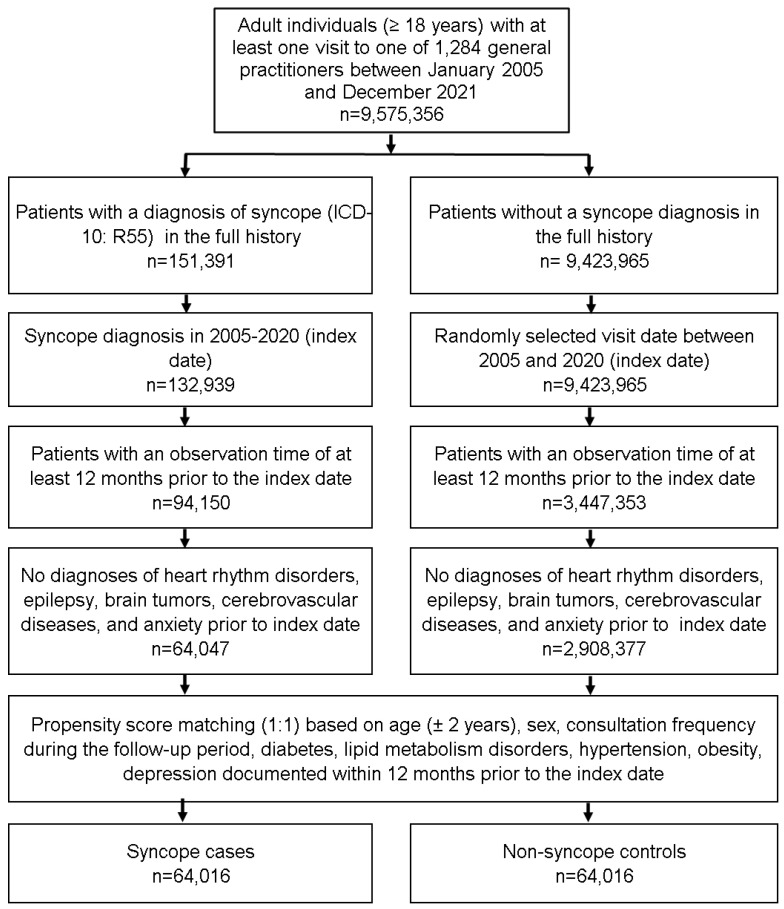
Selection of study patients.

This retrospective cohort study included adults aged ≥18 years who were diagnosed for the first time with syncope (International Classification of Diseases (ICD-10) code: R55) in one of 1284 general practices in Germany between January 2005 and December 2021 (index date). Patients were only included if they had an observation time of at least 12 months prior to the index date. Patients were excluded from the analysis if they had at least one diagnosis of ischemic stroke (ICD-10: I63, I64), including transient ischemic attack (TIA) (ICD-10: G45), arrhythmia (ICD-10: I47–I49), brain tumor (D32, D33, D42, D43, C70–C72), epilepsy or status epilepticus (ICD-10: G40, G41), and anxiety disorder (ICD-10: F41), prior to or on the index date. After applying similar inclusion criteria, patients without syncope were matched (1:1) to those with syncope using a propensity score based on age, sex, the number of consultations during the follow-up period (up to 6 months), and co-diagnoses documented within 12 months prior to and on the index date, including diabetes mellitus (ICD-10: E10–E14), hypertension (ICD-10: I10), lipid metabolism disorders (ICD-10: E78), obesity (ICD-10: E66), and depression (ICD-10: F32, F33). In people without syncope, the index date was a randomly selected visit date that occurred between January 2005 and December 2021. A flow chart that explains the selection of study participants is displayed in Figure 1.

### 2.3. Study Outcome

The target of this study was to determine the cumulative incidence of ischemic stroke/TIA, arrhythmia, brain tumor, epilepsy, and anxiety disorder within 6 months of the index date in patients with and without syncope. These diagnoses were known to be possible reasons for syncope, but they were also recognized as diagnoses often made by general practitioners.

### 2.4. Statistical Analyses 

Demographic and clinical characteristics of patients were compared for the cohorts with and without syncope using the Wilcoxon signed-rank test for continuous variables, the McNemar test for categorical variables with two categories, and the Stuart–Maxwell test for categorical variables with more than two categories. Finally, associations between syncope and incidences of ischemic stroke/TIA, arrhythmia, brain tumor, epilepsy, and anxiety disorder were investigated using multivariable logistic regression models adjusted for age, sex, and conditions documented within 12 months prior to or on the index date. Logistic regression was prioritized over Cox regression as no time to events (diagnoses); however, only the probabilities of the events were estimated. 

These models were applied to both the overall sample and individual age (i.e., ≤40, 41–50, 51–60, 61–70, and >70 years) and sex subgroups (i.e., female and male). The results of the logistic regression analyses are displayed as odds ratios (ORs) and 95% confidence intervals (CIs). Due to multiple comparisons, *p*-values lower than 0.001 were considered statistically significant. All analyses were performed using SAS 9.4 (SAS institute, Cary, NC, USA).

## 3. Results

### 3.1. Baseline Characteristics

The present retrospective cohort study included 64,016 patients with and 64,016 patients without syncope. The characteristics of participants after 1:1 matching are displayed in Table 1. The mean (standard deviation) age was 54.5 (20.9) years, and the proportion of women was 56.5%. Each patient visited the physician an average of 7.1 times during the follow-up period. Due to the matching process, the cohorts did not differ in terms of age, sex, visit frequency, or co-morbidities.

### 3.2. Cumulative Incidence of Outcome Diagnoses

**Figure 2:**
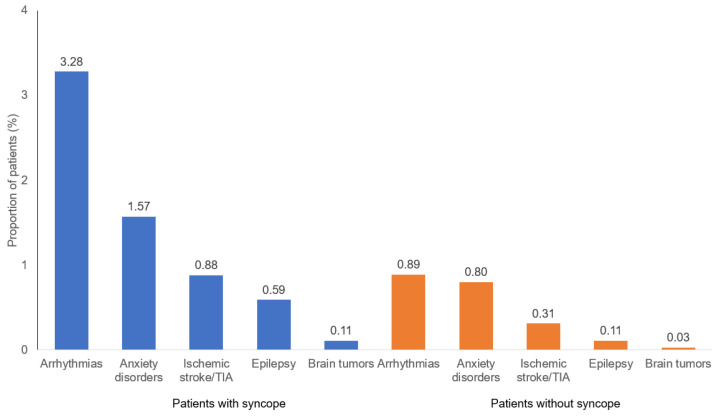
Six-month cumulative incidences of ischemic stroke, arrhythmia, brain tumor, epilepsy, and anxiety disorder in patents with and without syncope.

Figure 2 shows the cumulative incidence of the outcome diagnoses. In total, 6.43% of syncope patients were diagnosed with one of the five outcome diseases within 6 months of the index date. The most common diagnoses were arrhythmias (3.28%), followed by anxiety disorders (1.57%), ischemic stroke/TIA (0.88%), and epilepsy (0.59%). Brain tumors were rare (0.11%), and only two cases of malignant tumors were recorded. The proportion of non-syncope patients with at least one of the five diseases investigated was 2.14%.

### 3.3. Association between Syncope and Ischemic Stroke/TIA

The results of the logistic regression analyses are displayed in Table 2. There was a positive and significant association between syncope and incident ischemic stroke/TIA (OR = 2.83, 95% CI = 2.41–3.32) in the overall sample. This association was found to be strongest in the age group 41–50 years (OR = 11.95, 95% CI = 3.68–38.83), followed by the age group ≤ 40 years (OR = 6.60, 95% CI = 1.96–22.22). The association was similar between women (OR = 2.79, 95% CI = 2.24–3.48) and men (OR = 2.91, 95% CI = 2.29–3.69).

### 3.4. Association between Syncope and Arrhythmias

There was a positive and significant association between syncope and incident arrhythmias (OR = 3.81, 95% CI = 3.47–4.18), which was strongest among patients aged 51–60 (OR = 6.15, 95% CI = 4.57–8.28), followed by the age group 41–50 years (OR = 4.93, 95% CI = 3.44–7.07). The association had similar significance in women (OR = 3.90, 95% CI = 3.43–4.43) and men (OR = 3.71, 95% CI = 3.24–4.25) (Table 2).

### 3.5. Association between Syncope and Brain Tumors

There was a positive and significant association between syncope and brain tumors (OR = 4.24, 95% CI = 2.50–7.19). In the age-stratified analyses, this association was not significant in the age groups ≤ 40, 41–50, 51–60, and 61–70, only being significant among patients aged >70 (OR = 8.66, 95% CI = 2.62–28.63). This age group had the biggest absolute difference between cohorts (0.14% vs. 0.02%). The association was slightly stronger in women (OR = 4.64, 95% CI = 2.42–8.91) than in men (OR = 3.50, 95% CI = 1.41–8.67) (Table 2).

### 3.6. Association between Syncope and Epilepsy

We observed a strong positive association between syncope and subsequent epilepsy diagnosis (OR = 5.52, 95% CI = 4.27–7.14), which was strongest in the age group ≤ 40 (OR = 11.26, 95% CI = 5.89–21.51), followed by 51–60 years (OR = 7.72, 95% CI = 3.85–15.47), and 61–70 years (OR = 7.24, 95% CI = 3.45–15.19). The association had similar significance in women (OR = 5.38, 95% CI = 3.77–7.69) and men (OR = 5.67, 95% CI = 3.91–8.21) (Table 2).

### 3.7. Association between Syncope and Anxiety Disorders

Although the association between syncope and subsequent anxiety disorder diagnosis was positive and significant (OR = 1.99, 95% CI = 1.79–2.21), as well as being confirmed in each age group and observed in both women and men, this association was weaker than associations observed for other diagnoses (Table 2).

## 4. Discussion

### 4.1. Main Findings

In this retrospective cohort study, we found that a syncope was significantly associated with subsequent diagnoses of anxiety disorder, ischemic stroke/TIA, cardiac arrhythmia, brain tumor, and epilepsy. However, the cumulative incidence of any of the diseases was generally found to be low in the cohorts with and without a syncope diagnosis.

### 4.2. Interpretation of Findings

Syncope is a common condition in clinical practice, having mostly benign causes [1,10,12]. As previous studies have shown, adverse outcomes and increased mortality are mainly associated with the simultaneous presence of serious underlying conditions, especially cardiovascular diseases, and appear to be generally unrelated to the mechanism of syncope [37,38,39].

Nevertheless, it is possible that syncope is associated with a low-but-significant risk of adverse outcomes, including death. This association is due, first and foremost, to underlying cardiovascular conditions, which outlines why risk stratification has a significant impact on the clinical assessment of syncope [31,37,38,39]. Importantly, the majority of syncope cases have a favorable prognosis, as most common causes of syncope are benign, especially neurally mediated reflex syncope and syncope due to orthostatic hypotension [1,15]. Consequently, the presence of serious diseases is not expected in most cases of syncope, although thorough medical evaluation of the patient and the implementation of validated algorithms remain of great importance [16,40]. This view of syncope as tending to be rather benign in the majority of cases is also consistent with the findings of this retrospective cohort study, which includes a large cohort of more than 128,000 outpatients from general and specialized practices in Germany. 

Likewise, various studies have shown an increased incidence of psychiatric disorders, such as anxiety disorders, in patients with syncope compared to those without syncope [26,27,28]. However, the exact inclusion criteria for patients with syncope differed from that used in our study. For example, other studies only included patients with neurally mediated reflex syncope, whereas our study included syncope of different etiologies and also showed differences in the frequency of occurrence of syncopal spells, making it difficult to compare exact incidences. Nonetheless, the results of our study also confirm a relatively increased incidence of anxiety disorder in a cohort of patients with syncope compared to a cohort without the condition. However, of the five subsequent diagnoses studied, anxiety disorder showed the weakest association with a preceding syncope.

As was already the case for anxiety disorder, we also found that, in general, the absolute risk of developing one of the four remaining diagnoses included in this study after experiencing syncope is relatively low, although patients with previous syncope nevertheless show a significantly increased relative risk compared to persons without syncope.

In particular, it should be noted that the risk of brain tumor was low and only two of all brain tumors recorded in the 6-month period among patients diagnosed with syncope were malignant. Only slightly more than one in a thousand patients in the group with syncope were diagnosed with a brain tumor in the 6-month period, and this figure was even lower than one in three thousand in the group without syncope. These results are consistent with the findings of Lorenzen et al., who’s large population-based cohort study investigated whether syncope was a marker for hidden cancer, showing an increased but relatively low cumulative risk of brain tumor over a six-month period for a cohort of syncope patients [20]. 

As possible mechanisms of an association between brain tumors and syncope, the authors of the study identified, among other factors, compression of blood vessels, infiltration of the vagus and glossopharyngeal nerves, and infiltration of the brain areas relevant for cardiovascular functions, as well as tumor-induced anemia and tumor-induced venous thromboembolism, all of which can cause cerebral hypoperfusion and, thus, result in syncope [20]. Furthermore, the association between syncope and brain tumor diagnosis was only significant in the age groups 61–70 years and >70 years. A possible explanation for this age effect could be the already more common presence of risk factors for syncope with increasing age. More common cardiovascular multimorbidity, polypharmacy, and physiological changes in heart rate and blood pressure already increase susceptibility to syncope at an older age [41,42,43,44].

The subsequent diagnosis of ischemic stroke/TIA also showed a clear association with a previous syncope. Once again, the incidence was higher among patients with syncope than those without it, although the absolute incidences in both patient groups were comparatively low. One possible explanation for this association could be the presence of syncope with cardiac cause. Syncope is of cardiac origin in approximately one out of ten patients [15]. While cardiac causes of syncope, such as cardiac arrhythmias or structural heart disease, increase the risk of ischemic stroke/TIA, they are also risk factors for the occurrence of cardiac-related syncope [45,46,47,48,49].

Of the five diagnoses studied in the 6-month period, cardiac arrhythmias had the highest incidence among patients with and without syncope. This result is not surprising, since cardiac arrhythmias are common in the general population, with atrial fibrillation being the most common arrhythmia [50,51,52,53,54]. However, the data we evaluated do not allow further classification of the cardiac arrhythmias diagnosed during the 6-month period.

The most significant association with prior syncope diagnosis was shown for subsequent diagnosis of epilepsy. In this regard, however, it should be taken into account that various studies pointed out the diagnostic difficulties in distinguishing syncope from epilepsy [23,55,56,57].

For instance, Rangel et al. reported a proportion as high as one third of patients with syncope who had been misdiagnosed with epilepsy [57]. Although this study only included patients with refractory epilepsy, and, thus, examined a highly symptomatic cohort, [57] epilepsy has nevertheless been widely documented as a common misdiagnosis of syncope [23,55,56,57,58].

In view of the above information, it should be mentioned that a “diagnostic overlap” between syncope and epilepsy may also have occurred in this study; consequently, the possibility of misdiagnoses in this context cannot be excluded [57].

### 4.3. Clinical Implications and Directions for Future Research

Given that syncope is benign in the majority of cases, some serious medical conditions are relatively rare; however, as there is a non-negligible number of cases, the further development and improvement of clinical tools for risk stratification in syncope patients is of particular importance. This approach would reduce unnecessary diagnostics in low-risk patients, which are associated with higher costs for the health care system and health care facilities [11,59,60,61].

### 4.4. Strengths and Limitations

The major strengths of this study are the large sample size, the inclusion of different outcomes, and the matched-pair design. However, the findings of the present study must be interpreted in light of its limitations. Firstly, diagnoses, in our analyses, can be based on ICD-10 codes. ICD-10 codes do not allow the identification of the disease severity as well as syncope type (cardiac, vasovagal, orthostatic, neurologic and so on) and syncope duration. However, based on the first syncope documentation, physicians often cannot say for sure what type of syncope a patient experienced. As syncope type is very relevant as a possible symptom of severe disease, this limitation should always be noted when interpreting the results of this study. Secondly, the Disease Analyzer database does not provide data on lifestyle-related risk factors (e.g., smoking, alcohol consumption, and physical activity), which may potentially introduce bias to our analysis. Thirdly, no information was available regarding the methods of diagnosis, and it is possible that diagnoses recorded by the general practitioner in charge of a patient may have been made previously by a specialist or in a hospital setting without the GP being involved in the diagnostics process. As a consequence, no data are available regarding the type and degree of heart rhythm disturbances, type of epileptic seizures, localization of brain damage in stroke, or localization and type of brain tumor. Furthermore, outcome diagnoses mentioned in this study may have been diagnosed at other practices (e.g., psychiatry, neurology, or cardiology practices or in hospitals), and the incidences of these conditions may have been underestimated in the present study. Fourthly, we did not analyze therapies. Concomitant therapies, including anticoagulation drugs, antiepileptics, and neuroleptics or antidepressants, could also have influenced the results of this study. Finally, due to this study’s design, we can only make assumptions about associations between variables and cannot infer any causal relationships.

## 5. Conclusions

Syncope is significantly associated with an increased risk of subsequent ischemic stroke/TIA, cardiac arrhythmia, brain tumor, epilepsy, and anxiety disorder. However, the cumulative incidences of all five diagnoses are low in absolute terms in the cohorts with and without syncope. This result confirms the general view of syncope as being a relatively benign condition and, at the same time, emphasizes the importance of a thorough diagnostics process, as well as effective risk stratification for syncope patients to identify those individuals at increased risk of the occurrence of adverse outcomes.

## Figures and Tables

**Table 1:** Characteristics of study patients after 1.1 matching.

Variable	Syncope (n = 64,016)	No Syncope (n = 64,016)	Standard Mean Differnces
Age (in years)			
Mean (standard deviation)	54.5 (20.9)	54.5 (20.9)	0.00
≤ 40	28.6	28.6	0.10
41–50	12.5	12.5	
51–60	16.4	16.5	
61–70	14.3	14.5	
>70	28.1	28.0	
Sex			
Female	56.6	56.6	0.00
Male	43.6	43.6	
Number of consultations per year during the follow-up period; mean (standard deviation)	7.1 (4.3)	7.1 (4.3)	0.00
Conditions documented within 12 months prior to or on the index date			
Diabetes mellitus	15.4	15.4	0.00
Hypertension	41.7	41.7	0.00
Lipid metabolism disorders	28.6	28.6	0.00
Obesity	9.9	9.9	0.00
Depression	18.8	18.8	0.00

Data are percentages unless otherwise specified.

**Table 2:** Association between syncope and subsequent stroke, arrhythmia, brain tumor, epilepsy, and anxiety disorder within 6 months of syncope diagnosis in patients who were followed in general practices in Germany (multivariable logistic regression models).

Population *	Proportion (%) in Patients with Syncope	Proportion (%) in Patients without Syncope	OR (95% CI)	*p*-Value
*Ischemic stroke/TIA*				
Overall **	0.88	0.31	2.83 (2.41–3.32)	<0.001
≤40 ***	0.11	0.02	6.60 (1.96–22.22)	<0.001
41–50 ***	0,45	0.04	11.95 (3.68–38.83)	<0.001
51–60 ***	0.69	0.22	3.20 (2.00–5.12)	<0.001
61–70 ***	1.26	0.36	3.61 (2.45–5.29)	<0.001
>70 ***	1.78	0.78	2.32 (1.90–2.83)	<0.001
Female ****	0.82	0.30	2.79 (2.24–3.48)	<0.001
Male ****	0.96	0.33	2.91 (2.29–3.69)	<0.001
*Arrhythmia*				
Overall **	3.28	0.89	3.81 (3.47–4.18)	<0.001
≤40 ***	1.45	0.38	3.82 (2.94–4.98)	<0.001
41–50 ***	2.18	0.45	4.93 (3.44–7.07)	<0.001
51–60 ***	2.91	0.48	6.15 (4.57–8.28)	<0.001
61–70 ***	3.78	0.96	4.08 (3.22–5.16)	<0.001
>70 ***	5.59	1.80	3.23 (2.84–3.66)	<0.001
Female ****	3.12	0.82	3.90 (3.43–4.43)	<0.001
Male ****	3.49	0.97	3.71 (3.24–4.25)	<0.001
*Brain tumor*				
Overall **	0.11	0.03	4.24 (2.50–7.19)	<0.001
≤40 ***	0.04	0.01	3.48 (0.72–16.76)	0.120
41–50 ***	0.14	0.06	2.21 (0.77–6.36)	0.143
51–60 ***	0.16	0.05	3.43 (1.26–9.29)	0.016
61–70 ***	0.12	0.02	5.55 (1.23–25.06)	0.026
>70 ***	0.14	0.02	8.66 (2.62–28.63)	<0.001
Female ****	0.14	0.03	4.64 (2.42–8.91)	<0.001
Male ****	0.08	0.02	3.50 (1.41–8.67)	0.007
*Epilepsy*				
Overall **	0.59	0.11	5.52 (4.27–7.14)	<0.001
≤40 ***	0.61	0.05	11.26 (5.89–21.51)	<0.001
41–50 ***	0.44	0.12	3.52 (1.74–7.11)	<0.001
51–60 ***	0.66	0.09	7.72 (3.85–15.47)	<0.001
61–70 ***	0.62	0.09	7.24 (3.45–15.19)	<0.001
>70 ***	0.59	0.18	3.31 (2.23–4.91)	<0.001
Female ****	0.53	0.10	5.38 (3.77–7.69)	<0.001
Male ****	0.67	0.12	5.67 (3.91–8.21)	<0.001
*Anxiety disorder*				
Overall **	1.57	0.80	1.99 (1.79–2.21)	<0.001
≤40 ***	1.84	0.87	2.14 (1.77–2.58)	<0.001
41–50 ***	2.18	0.92	2.39 (1.82–3.14)	<0.001
51–60 ***	1.67	0.75	2.25 (1.72–2.94)	<0.001
61–70 ***	1.21	0.72	1.68 (1.24–2.28)	<0.001
>70 ***	1.14	0.73	1.59 (1.27–1.98)	<0.001
Female ****	1.75	0.92	1.91 (1.67–2.19)	<0.001
Male ****	1.34	0.63	2.13 (1.78–2.55)	<0.001

Abbreviations: OR, odds ratio; CI, confidence interval. * Populations are the subgroups in which the associations between syncope and the defined diagnoses were investigated. Populations are not variables based on which associations with syncope or defined diagnoses were analyzed. ** adjusted for age, sex, diabetes mellitus, hypertension, lipid metabolism disorders, obesity, and depression. *** adjusted for sex, diabetes mellitus, hypertension, lipid metabolism disorders, obesity, and depression. **** adjusted for age, diabetes mellitus, hypertension, lipid metabolism disorders, obesity, and depression.

## Data Availability

The datasets used and analyzed during the current study are available from the corresponding author on reasonable request.
